# Topological patterns of motor networks in Parkinson’s disease with different sides of onset: A resting-state-informed structural connectome study

**DOI:** 10.3389/fnagi.2022.1041744

**Published:** 2022-10-26

**Authors:** Xiuli Zhang, Ruohan Li, Yingying Xia, Houliang Zhao, Lulu Cai, Jingyun Sha, Qihua Xiao, Jie Xiang, Chao Zhang, Kai Xu

**Affiliations:** ^1^Department of Radiology, Affiliated Hospital of Xuzhou Medical University, Xuzhou, Jiangsu, China; ^2^School of Medical Imaging, Xuzhou Medical University, Xuzhou, Jiangsu, China; ^3^Department of Neurology, Affiliated Hospital of Xuzhou Medical University, Xuzhou, Jiangsu, China; ^4^Department of Rehabilitation, Affiliated Hospital of Xuzhou Medical University, Xuzhou, Jiangsu, China

**Keywords:** Parkinson’s disease, graph theory, fMRI, diffusion tensor imaging, resting state

## Abstract

Parkinson’s disease (PD) has a characteristically unilateral pattern of symptoms at onset and in the early stages; this lateralization is considered a diagnostically important diagnosis feature. We aimed to compare the graph-theoretical properties of whole-brain networks generated by using resting-state functional MRI (rs-fMRI), diffusion tensor imaging (DTI), and the resting-state-informed structural connectome (rsSC) in patients with left-onset PD (LPD), right-onset PD (RPD), and healthy controls (HCs). We recruited 26 patients with PD (13 with LPD and 13 with RPD) as well as 13 age- and sex-matched HCs. Rs-fMRI and DTI were performed in all subjects. Graph-theoretical analysis was used to calculate the local and global efficiency of a whole-brain network generated by rs-fMRI, DTI, and rsSC. Two-sample *t*-tests and Pearson correlation analysis were conducted. Significantly decreased global and local efficiency were revealed specifically in LPD patients compared with HCs when the rsSC network was used; no significant intergroup difference was found by using rs-fMRI or DTI alone. For rsSC network analysis, multiple network metrics were found to be abnormal in LPD. The degree centrality of the left precuneus was significantly correlated with the Unified Parkinson’s Disease Rating Scale (UPDRS) score and disease duration (*p* = 0.030, *r* = 0.599; *p* = 0.037, *r* = 0.582). The topological properties of motor-related brain networks can differentiate LPD and RPD. Nodal metrics may serve as important structural features for PD diagnosis and monitoring of disease progression. Collectively, these findings may provide neurobiological insights into the lateralization of PD onset.

## Introduction

Parkinson’s disease (PD) has a characteristically asymmetrical pattern of onset, manifesting as unilateral motor abnormalities, and the symptoms can remain lateralized throughout the disease course (Djaldetti et al., 2006; Verreyt et al., 2011; Elkurd et al., 2021). Unilateral motor symptoms can help differentiate PD from atypical PD syndrome (Hughes et al., 1992). Left-onset PD (LPD) and right-onset PD (RPD) differ in their progression and risk of complications (Baumann et al., 2014). In particular, a recent study has concluded that patients with LPD may have greater motor and non-motor symptoms but a better quality of life than those with RPD (Cubo et al., 2020). Patients with LPD and RPD respond differently to levodopa and rehabilitation treatments in some cognitive domains (Hanna-Pladdy et al., 2015; Ortelli et al., 2018). An improved understanding of the onset of asymmetric symptoms could aid in the early diagnosis of PD and the monitoring of disease progression, which may enable personalized treatment of PD in the future (Feis et al., 2015). However, it is worth noting that the mechanism that causes the asymmetry of motor symptoms in PD has not yet been elucidated.

Numerous studies have found that PD is a brain network disorder (Canu et al., 2015; Wang et al., 2016; Ji et al., 2018). In the early stages of PD, the accumulation and dissemination of α-synuclein aggregates in the brain are associated with alterations in resting-state functional connectivity (FC) in the basal ganglia and intercortical and intracortical networks (Tessitore et al., 2019). Resting-state functional MRI (rs-fMRI) studies have also provided evidence that motor and non-motor symptoms of PD are closely related to the disruption of brain structural and FC (Yang et al., 2021). The current perspective suggests that the asymmetric motor symptoms of PD may be the result of unequal degeneration of midbrain dopaminergic neurons (Li et al., 2020a); however, it remains difficult to explain the relationship between PD-related brain network alterations and clinical manifestations.

The brain is a complex network with functional integration and separation (Shirer et al., 2012). Rs-fMRI is an established tool to non-invasively and effectively explore intrinsic brain activity, and it has been widely used to investigate neurological diseases (Yagi et al., 2010; Li et al., 2020b). Recently, various approaches based on rs-fMRI have been employed to explore the mechanism of PD; such approaches include spontaneous local brain activity, amplitude of low-frequency fluctuation, regional homogeneity (Li et al., 2020a,b), FC between remote brain regions, and graph-theory-based approaches to clarify the properties of the brain connectome (Kim et al., 2017; Tang et al., 2018). PD patients have been demonstrated to have FC changes in multiple brain regions, such as decreased FC between the premotor cortex and the putamen, increased FC between the primary motor cortex and the cerebellum (Wu et al., 2010), decreased cortico-striatal-thalamic FC (Hacker et al., 2012) and increased FC between the primary motor cortex and the subthalamic nucleus (Baudrexel et al., 2011). Decreased FC indicates disease progression, and increased FC may suggest compensation or remodeling of brain function (Agosta et al., 2014; Ji et al., 2018). Few previous fMRI-based studies have separated LPD from RPD when exploring abnormal brain network connectivity.

Graph-theory-based approaches are a new method of brain network analysis that can quantitatively describe the characteristics of brain connectivity (Sporns, 2018). A previous study using a graph-theory-based approach found that the topological properties of brain networks were disrupted in PD patients; the ability to identify these disruptions can be helpful in diagnosing PD (Kim et al., 2017), evaluating disease progression and monitoring the effects of treatment (Suo et al., 2017; Huang et al., 2020). By investigating the network topological properties of patients with different degrees of cognitive impairment in PD, researchers found that the topological organization of the networks was gradually destroyed; this degradation is a diagnostically useful sign of prodromal PD dementia (Lopes et al., 2017). Comparing the topology of the brain functional connectome between PD patients and healthy controls (HCs) may provide new insight into the pathophysiological mechanisms of PD (Prajapati and Emerson, 2021). However, previous graph-theoretical analysis of PD combined patients with LPD and RPD into a single group to compare them with HCs, whereas little extant research has focused on differences in network properties between PD patients with different sides of onset and HCs.

Functional-by-structural hierarchical (FSH) mapping is a novel method that integrates rs-fMRI and diffusion tensor imaging (DTI) data into a single functional-structural network; this method can improve sensitivity for detecting network-level vulnerabilities in people with subtle age-related cognitive decline before the onset of overt cognitive impairment (Korthauer et al., 2018). It remains unknown whether FSH mapping is sensitive enough to detect brain network differences among LPD patients, RPD patients and HCs. Therefore, the purpose of the present investigation was to compare the graph-theoretical properties of a whole-brain network generated by using rs-fMRI, DTI, and the resting-state-informed structural connectome (rsSC) in patients with LPD, patients with RPD, and HCs. This study is motivated by an interest in discovering how these connectivity measures can help clarify the underlying pathogenesis of LPD and RPD.

## Materials and methods

### Subjects

This study was approved by the local ethical committee of Xuzhou Affiliated Hospital, Xuzhou Medical University. In compliance with the Declaration of Helsinki, written informed consent was obtained from all subjects before participation.

The project used a convenience sample of 32 right-handed hospitalized PD patients (17 with LPD and 15 with RPD) who met the diagnostic criteria for PD as set forth by the UK Parkinson’s Disease Society Brain Bank (Hughes et al., 1992). All patients underwent a comprehensive clinical assessment, including the Unified Parkinson’s Disease Rating Scale (UPDRS), Hoehn and Yahr (H-Y) staging, the Montreal Cognitive Assessment (MoCA), and the Mini-Mental State Examination (MMSE). All the patients underwent routine medical treatment, and none of them received any other relevant interventions. Additionally, all patients were free of cognitive impairment. The exclusion criteria for the participants included MRI-confirmed brain abnormalities (trauma, stroke, tumor, and infection) and MRI contraindications (claustrophobia and implanted metal parts). In addition, subjects with a history of drug abuse, alcohol abuse, or syncope were excluded. Fifteen right-handed age- and sex-matched healthy volunteers were included as HCs. All the patients underwent neuropsychological tests and fMRI scans in their off-medication state, and HCs were examined according to the same protocol.

### Magnetic resonance imaging data acquisition

All participants were scanned in a 3.0-tesla MRI scanner (GE Medical Systems, Signa HD, Waukesha, WI, USA) with an eight-channel head coil. During MRI scanning, comfortable foam pads were used to stabilize the head of each subject to minimize head motion, and all the subjects wore earplugs to reduce noise from the MRI machine. A 3D-T1 brain volume (BRAVO) sequence was used to acquire high-resolution T1-weighted images, providing isotropic voxels that measured 1 mm × 1 mm × 1 mm. Then, an echo-planar imaging sequence was employed to acquire resting blood oxygen level-dependent images. The parameters of the protocol were as follows: repetition time (TR) = 2,000 ms; echo time (TE) = 30 ms; field of view = 220 mm × 220 mm; slice thickness = 3 mm; slice gap = 1 mm; voxel size = 3.4 mm × 3.4 mm × 4.0 mm; 36 slices; flip angle = 90°; total number of volumes per subject, 185. DTI data were obtained using the following parameters: TR/TE = 9,000/90 ms, matrix = 128 mm × 128 mm, field of view = 256 mm × 256 mm, number of diffusion gradient directions = 64, b value = 1,000, voxel size 3 mm × 2 mm × 2 mm.

### Preprocessing of resting-state functional MRI and diffusion tensor imaging data

A graph-theoretical network analysis toolbox for imaging connectomics (GRETNA)^1^ was used to perform data preprocessing (Wang et al., 2015). The main steps were as follows: (1) the first 10 time points from each subject were removed; (2) slice-timing correction was used to correct time differences on the remaining 175 volumes; and (3) realignment was used to correct individual-level head motion through Friston’s 24-parameter model, and any subject with maximum head displacement >2 mm, maximum rotation >2.0°, or mean framewise displacement (FD) > 0.3 was excluded from the study (Yan et al., 2013; Zhang et al., 2020). To further minimize the potential influence of head motion, mean FD was set as a covariate for further group-level statistics (Satterthwaite et al., 2012; Zeng et al., 2014). Subsequently, individual structural images were coregistered to the mean functional image, and then the transformed structural images were segmented into gray matter, white matter (WM) and cerebrospinal fluid. Diffeomorphic anatomical registration through exponentiated Lie algebra (DARTEL) was performed to estimate the normalization parameters from individual native space to Montreal Neurological Institute (MNI) space (Ashburner, 2007). After spatial normalization, functional images were resampled at a voxel size of 2 mm × 2 mm × 2 mm. The functional volumes were spatially smoothed with a 6-mm full-width-at-half-maximum Gaussian kernel.

Diffusion tensor imaging data in the Digital Imaging and Communications in Medicine (DICOM) format were processed on a Linux workstation. The software Pipeline for Analyzing braiN Diffusion imAges (PANDA) was employed for fully automated processing of brain diffusion images (Cui et al., 2013). The main steps of the process were as follows: (1) converting DICOM data to the NIFTI format; (2) eddy current correction; (3) estimating brain masks; (4) cropping images; and (5) averaging acquisitions and calculating DTI metrics. The resultant warping transformations were then used to resample the images of the diffusion metric of interest (fractional anisotropy, FA) into the MNI space with a customized spatial resolution of 2 mm × 2 mm × 2 mm. Network nodes were defined according to the AAL90 atlas (Ham et al., 2015). Finally, deterministic tractography was performed to obtain the FA matrix, with the following parameters: FA threshold = 0.2–1, angle threshold = 45°.

### Network construction of the brain

Edges and nodes are the basic elements of a brain network, where each node represents a brain region and each edge describes the connectivity between two brain regions. We employed GRETNA software to determine the FC, structural connectivity, and functional-structural matrices on the basis of brain images (Chung et al., 2019). For the construction of the FC network, each node was defined using the AAL90 brain atlas template, and each region was considered a node to create the connectivity matrix. The edges were calculated using Pearson correlation coefficients of the mean time series obtained from each node. Finally, the 90 × 90 FC matrix was determined for each subject. The definition of each node of the structural network was also using the AAL90 brain atlas template, and the edges of the structural network were defined as WM fiber between each node, which were constructed utilizing deterministic fiber tracing technology. Similarly, the 90 × 90 WM matrix was determined for each subject.

The sparsity threshold was applied to create the binary matrix (adjacency matrix). The sparsity threshold denotes the ratio of the number of actual edges to the total number of possible edges in the fMRI matrix. We used threshold values ranging from 0.05 to 0.5 within 0.05 intervals to remove the possible false edges of the fMRI matrix. We chose an FA threshold of 0, leaving the DTI matrix unchanged. Next, the fMRI and DTI matrices were *z*-transformed before being input into the FSH mapping pipeline.

### Functional-by-structural hierarchical mapping

The FSH mapping pipeline has been described in detail in previous works in the literature (Leow et al., 2012; Ajilore et al., 2014; Korthauer et al., 2018). Briefly, FSH mapping employs simulated annealing to find the optimal utilization matrix to maximize the observed goodness-of-fit between rs-fMRI and rsSC. Using a randomly chosen starting seed, calculate the probability distribution to create the optimal solution U for each group. A Weighted rsSCs were then created by multiplying each participant’s structural connectivity matrix S by the binarized group U matrix. In the current study, FSH mapping was used to integrate the functional data (in the form of FC) and structural data (in the form of FA) into a single graph and generate rsSC. WM fiber connectivity and resting-state brain FC are not intended to be equivalent; resting-state FC can correspond to either direct or indirect neuroanatomical WM connections between two brain regions. FSH mapping assumes that higher levels of rs-fMRI correlation reflect stronger structural interactions, and FC data may be used to infer the underlying pattern of WM engagement that occurs during this particular resting state. The resulting rsSC in this study reflects the FA network underlying the observed functional connectome.

### Statistical analysis

A chi-square test was employed to test for significant differences in gender distribution, and a one-way ANOVA test was used to observe the age difference among the three groups. For the LPD and RPD groups, a two-sample *t*-test was performed to analyze intergroup differences in disease duration, UPDRS scores, H-Y stages, MoCA scores, and MMSE scores. The statistical analysis described above was performed in SPSS version 28.0 (SPSS Inc., Chicago, IL, USA). These group differences in global and nodal metrics were compared through a two-sample *t*-test by using the GRETNA toolbox, and the results were corrected for multiple comparisons using the false discovery rate (FDR) correction with a threshold of *p* < 0.05. To further observe the relationship between brain network changes and clinical symptoms, Pearson correlation analysis was conducted between network metrics and clinical scale scores, including UPDRS scores, H-Y stages, MoCA scores, and MMSE scores.

## Results

### Demographics of the participants

Six patients and four healthy volunteers were excluded due to obvious head movement or failure to complete the scored evaluations. Ultimately, 13 LPD patients (7 males and 6 females, 60.62 ± 6.39 years old), 13 RPD patients (8 males and 5 females, 59.15 ± 6.26 years old), and 13 healthy volunteers (5 males and 8 females, 61.42 ± 7.42 years old) were included in the present study. No significant difference was found in age (*p* = 0.72) or gender (*p* = 0.45) among the LPD, RPD, and HC groups. There was also no difference in PD duration, UPDRS scores, H-Y stages, MoCA scores, or MMSE scores between the LPD and RPD groups (*p* > 0.05) (Table 1).

**TABLE 1:** Demographics and clinical data.

Variable	HCs (*N* = 13)	LPD (*N* = 13)	RPD (*N* = 13)	*p*
Gender (M/F)	5/8	7/6	8/5	0.45^#^
Age (years)	61.42 ± 7.42	60.62 ± 6.39	59.15 ± 6.26	0.72*
Duration of PD (years)	N/A	3.92 ± 1.85	3.6 ± 1.76	0.67^&^
UPDRS-III score	N/A	24.08 ± 6.76	24.53 ± 6.31	0.28^&^
H-Y stage	N/A	1.65 ± 0.80	1.84 ± 0.66	0.27^&^
MoCA score	N/A	25.15 ± 2.15	24.04 ± 2.81	0.86^&^
MMSE score	N/A	27.9 ± 1.12	27.38 ± 1.33	0.51^&^

PD, Parkinson’s disease; HCs, healthy controls; M, male; F, female; UPDRS-III, Unified Parkinson’s Disease Rating Scale; H-Y, Hoehn and Yahr; MoCA, Montreal Cognitive Assessment; MMSE, Mini-Mental State Examination.

Data are presented as range and mean ± SD.

^#^The *p*-value was obtained using a chi-square test.

*The *p*-value was obtained using a one-way ANOVA test.

^&^The *p*-value was obtained using a two-sample *t*-test.

### Group analysis of global network metrics

**FIGURE 1:**
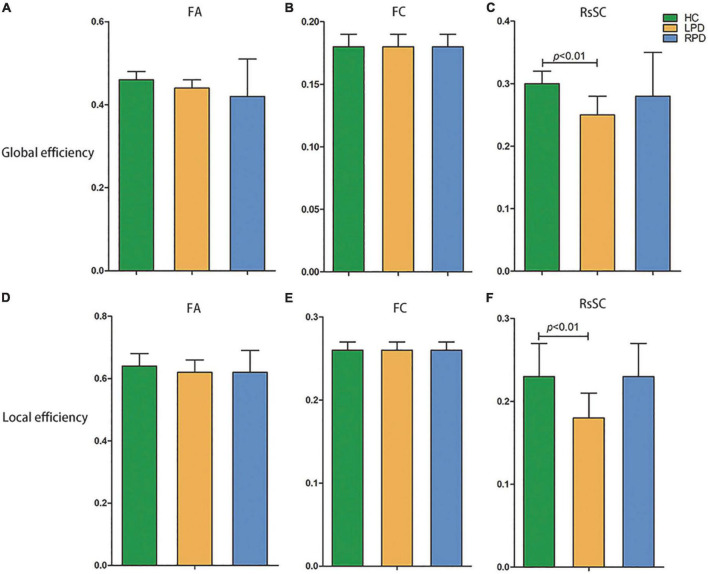
Decreased global efficiency **(C)** and local efficiency **(F)** were revealed in left-onset PD (LPD) patients compared with healthy controls (HCs) when the resting-state-informed structural connectome (rsSC) network was used, but no difference in global or local efficiency was revealed between LPD and right-onset PD (RPD) or between RPD and HCs. In the comparison fMRI (FC) and DTI (FA) network metrics, no significant difference was found in global or local efficiency among the LPD, RPD and HC groups **(A,B,D,E)**. FA, fractional anisotropy; FC, functional connectivity.

All three groups of subjects (LPD patients, RPD patients, and HCs) showed small-world properties (Supplementary Figure 1). In the comparison fMRI and DTI network metrics, no significant difference was found in global or local efficiency between the LPD and HC groups or between the RPD and HC groups. However, the rsSC network revealed significantly decreased global and local efficiency in LPD patients compared with HCs, while no difference in global or local efficiency was revealed between the LPD and RPD groups or between the RPD and HC groups (Figure 1).

### Group analysis of nodal network metrics in motor-related brain regions

The purpose of this study was to investigate the topological pattern of the motor-related networks on the side of PD onset; therefore, we chose the motion-related network for further analysis based on the Brodmann area (BA) template as well as the AAL template (Table 2).

**TABLE 2:** Coordinates and abbreviations for the network nodes of the automated anatomical labeling (AAL) atlas.

Nodal metrics	Regions	Abbreviation	MNI coordinates			Brodmann area (BA)
			*X*	*Y*	*Z*	
Betweenness centrality	Superior occipital gyrus	SOG.L	−16	−84	28	BA7
		SOG.R	24	81	30	BA7
	Middle occipital gyrus	MOG.L	−32	−81	16	BA7
		MOG.R	37	−80	19	BA7
Degree centrality	Superior occipital gyrus	SOG.L	−16	−84	28	BA7
	Rolandic operculum	ROL.R	53	−6	14	BA6
	Supplementary motor area	SMA.R	8	0	62	BA4/6/8
	Precentral gyrus	PreCG.L	−5	−43	25	BA4/6
	Rolandic operculum	ROL.R	53	−6	14	BA6
	Precuneus	PCUN.L	−7	−56	48	BA4/5/7
	Precuneus	PCUN.R	10	−56	44	BA4/5/7
Nodal efficiency	Median cingulate and paracingulate gyri	DCG.L	−5	−15	42	BA6
	Precentral gyrus	PreCG.L	−5	−43	25	BA4/6
	Paracentral lobule	PCL.L	−7	−25	71	BA4/6
	Precuneus	PCUN.L	−7	−56	48	BA5/7
	Supplementary motor area	SMA.R	8	0.2	62	BA4/6/8
	Paracentral lobule	PCL.R	7	−31	68	BA4
	Superior occipital gyrus	SOG.L	−16	−84	28	BA7

**FIGURE 2:**
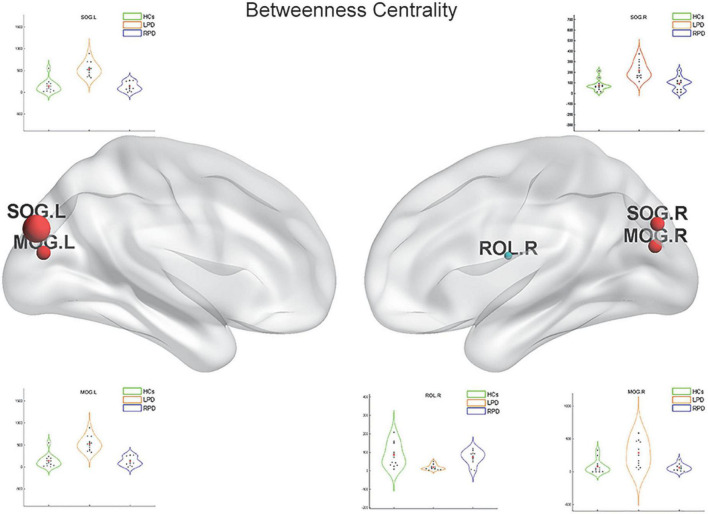
Left-onset PD (LPD) patients showed increased betweenness centrality (BC) in the bilateral superior occipital gyrus and bilateral middle occipital gyrus as well as decreased BC in the right Rolandic operculum.

**FIGURE 3:**
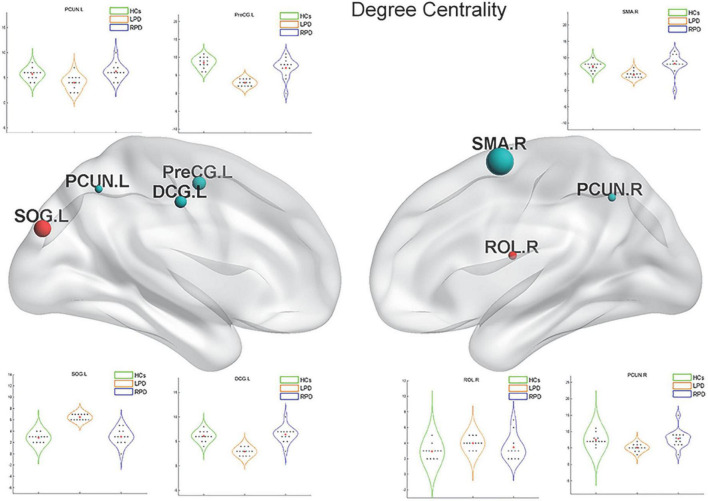
Left-onset PD (LPD) patients showed decreased degree centrality (DC) in the left median cingulate and paracingulate gyri, right supplementary motor area, left precentral gyrus, right Rolandic operculum, and bilateral precuneus as well as increased DC in the left superior occipital gyrus.

**FIGURE 4:**
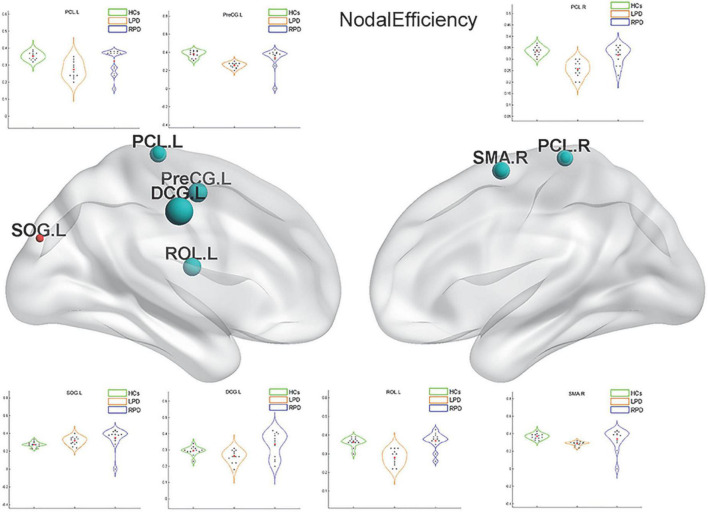
Left-onset PD (LPD) patients showed decreased nodal efficiency (NE) in the left median cingulate and paracingulate gyri, left precentral gyrus, bilateral paracentral lobule, and right supplementary motor area as well as increased NE in the left superior occipital gyrus.

Upon fMRI and DTI network analysis, we found no significant difference in the nodal network metrics when LPD and RPD patients were compared with HCs. Upon rsSC network analysis, we found significant intergroup differences in multiple network metrics (Table 2). Upon BC analysis, LPD patients showed increased BC in the bilateral superior occipital gyrus (SOG) and bilateral middle occipital gyrus (MOG) and decreased BC in the right Rolandic operculum (ROL.R) (Figure 2). In the DC comparison, LPD patients showed decreased DC in the left median cingulate and paracingulate gyri (DCG.L), right supplementary motor area (SMA.R), left precentral gyrus (PreCG.L), ROL.R, and bilateral precuneus (PCUN) as well as increased DC in the left SOG (SOG.L) (Figure 3). In the NE study, LPD patients showed decreased NE in the DCG.L, left PreCG (PreCG.L), bilateral paracentral lobule (PCL), and SMA.R as well as increased NE in the SOG.L (Figure 4). However, RPD patients did not show obvious differences in nodal network metrics compared with HCs.

**FIGURE 5:**
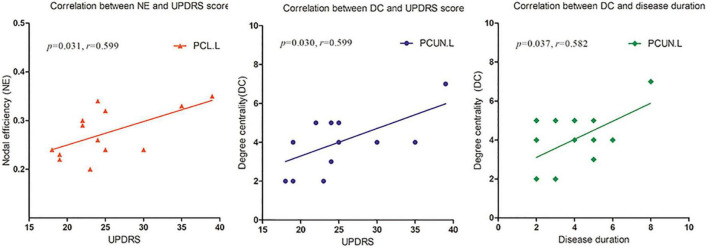
The nodal efficiency (NE) of left paracentral lobule (PCL.L) and degree centrality (DC) of the left precuneus (PCUN.L) were significantly correlated with Unified Parkinson’s Disease Rating Scale (UPDRS) scores (*p* = 0.031, *r* = 0.599; *p* = 0.030, *r* = 0.599). The DC of PCUN.L was also found to be significantly associated with disease duration (*p* = 0.037, *r* = 0.582).

Upon correlation analysis, we found that the NE of the PCL.L and the DC of the PCUN.L were significantly correlated with UPDRS scores (*p* = 0.031, *r* = 0.599; *p* = 0.030, *r* = 0.599). In addition, the DC of the PCUN.L was found to be significantly associated with disease duration (*p* = 0.037, *r* = 0.582) (Figure 5). No other significant correlation was revealed between network metrics and clinical scores.

## Discussion

We investigated the topological properties of the functional and structural connectomes of PD patients with different sides of onset. The property of small-worldness was present in PD. The current study found the following on the basis of the rsSC network: (1) significant differences in brain network metrics were detectable among patients with different sides of onset and HCs when graph-theoretical measures were used to characterize the properties of rsSC, even though no difference was evident from the rs-fMRI- or DTI-derived network alone; (2) significantly decreased global efficiency and local efficiency were evident in LPD patients; (3) compared with HCs, patients with LPD showed obvious abnormalities at the nodal level, such as decreased DC/NE and increased BC in multiple motor-related networks; and (4) certain brain regions were significantly associated with clinical conditions, including disease duration and UPDRS scores. These findings may provide neurobiological insights into the lateralization of the onset of PD.

Graph-theoretical indices have been considered to be more sensitive than traditional region of interest (ROI)–based analysis for detecting early-stage brain aberrations (Bullmore and Sporns, 2009). The brain has properties of functional separation and integration. Brain network integration can be measured using global efficiency, which is the average inverse shortest path length in the network. Local efficiency is defined by evaluating which of a node’s neighbors are neighbors of each other and is a measure of network segregation. The accumulation and dissemination of α-synuclein aggregates in the brain in the early stages of PD has been demonstrated to be associated with alterations in resting-state FC in the basal ganglia, intercortical, and intracortical networks (Stanziano et al., 2021). It has also been demonstrated that brain structural and functional connections are damaged in the early stages of PD. A previous rs-fMRI study of 54 PD patients found that the global efficiency of the motor network was decreased in PD patients compared with tremor patients and HCs (Novaes et al., 2021). Another rs-fMRI study showed that the global and local efficiency of the sensorimotor network and the visual network were disrupted in PD (Fang et al., 2017). In addition, a study of 23 early-stage PD patients showed that these patients had reduced global efficiency and segregation of structural brain network information processing compared with HCs (Vriend et al., 2018). In our study, rsSC networks showed that global and local efficiency were significantly reduced in patients with LPD; this finding is consistent with previous studies. On the other hand, we found no group difference in global or local efficiency generated through the rs-fMRI or DTI network alone, which may be because the sample size was too small to provide sufficient statistical power; however, this also demonstrates that rsSC is more sensitive than either rs-fMRI or DTI alone in identifying abnormal brain network properties in patients with PD (Korthauer et al., 2018). Furthermore, unlike previous studies, our study delineated the brain networks of LPD and RPD separately, and only patients with LPD were revealed to have abnormal network topology.

To observe local separation of brain function, three nodal metrics of the motor-related networks, namely, the DC, BC, and NE, were compared between groups by using rsSC. The nodal metrics for all rs-fMRI and DTI connectomes were calculated over the sparsity threshold. DC and BC can measure how important a node is within a network, that is, the centrality of a node, while NE is mainly related to short-range connections, which represent the capacity for local integration and transmission of information. Compared with HCs, LPD patients showed rsSC abnormalities in the motor-related networks, including the primary motor cortex (bilateral PCL), somatosensory association cortex (PreCG.L and DCG.L), and SMA. The most significant reductions in BC and DC were found in the primary motor cortex, SMA, PreCG, and bilateral PCUN, which are closely related to various motor functions (Ugur et al., 2005; Ikeda, 2007; Thibes et al., 2017; Bhattacharjee et al., 2021). Previous studies have demonstrated that stimulation of the primary motor cortex can significantly reduce the slowing and loss of motor function in patients with PD (Qiu et al., 2019). The current study found the BC and DC of the PCL.L simultaneously decreased in patients with LPD; additionally, the NE of the PCL.L was revealed to be closely correlated with the UPDRS score. The current findings indicated that the NE of the PCL.L can quantify motor dysfunction in patients with PD, which may provide theoretical support for future targeted therapy.

The PreCG.L is an important cortical projection region in the striato-thalamo-cortical (STC) loop and is responsible for the planning, initiation, and execution of exercise. The STC forms the neural network basis of PD bradykinesia, myotonia, and resting tremor (DeLong et al., 1984; Alexander et al., 1986; Lewis et al., 2007) as part of its involvement in PD (Stankovic et al., 2014). In addition, the SMA, one of the major components of the sensorimotor STC loop, rapidly evaluates successful and erroneous actions and thus mediates action monitoring (Bonini et al., 2014). In the present study, the LPD group exhibited nodal property alterations in the regions of the STC loop. Decreased nodal centrality and local information processing efficiency in these motor-related areas indicated their reduced roles in the motor-related networks of patients with PD. In addition, we found that the NE of the PreCG.L decreased, which means that there were fewer short-range connections in the PreCG.L and that the capacity for local integration of information was disrupted. The PreCG is an important portion of the cerebello-thalamo-cortical (CTC) loop. This circuit connects regions of the cerebellar cortex with the cerebral cortex. Lateral portions of the cerebellar cortex send projections via the dentate nucleus to the thalamus, which, in turn, projects to specific cortical areas. The CTC loop is generally considered a compensatory network, capable of offsetting the ill effects of STC degradation on motor regulation (Martinu and Monchi, 2013). The disrupted efficiency of the PreCG may be the result of this effect.

The DC of the bilateral PCUN in patients with LPD was decreased. The PCUN located mainly in the medial and posterior parietal lobe, consumes 35% more glucose than other brain regions in the resting state (Gusnard et al., 2001). According to previously published reports by our group and others, the PCUN.L is a vulnerable area, and it is closely associated with motor and non-motor symptoms of PD (Thibes et al., 2017; Jia et al., 2018; Zhang et al., 2019). Furthermore, we found that the DC of the PCUN.L was significantly related to the UPDRS score and disease duration. Thus, DC can not only directly reflect disruption of the PCUN.L but also quantify the degree of motor function impairment. Our study once again proved the importance of the PCUN.L in PD, and this region may be useful as a potential imaging feature to diagnose PD and monitor its progression.

Interestingly, we found that LPD patients showed increased BC in several regions of the occipital lobe, such as the bilateral SOG and MOG. The occipital lobe is the visual information processing center, which contains most of the visual cortex–related areas and participates in the transmission and reception of most visual information. The occipital lobe was demonstrated to be a vulnerable structure in studies of spontaneous brain activity and brain morphology (Uribe et al., 2016; Chen et al., 2022). The regional brain activity and volume of the occipital lobe were found to decrease in PD patients. However, other studies showed that the SOG and MOG had greater activity and volume in PD patients than in HCs (Choe et al., 2013; Li et al., 2016), which might reflect compensation for the disruption of visuospatial ability in PD. Increased nodal centrality in these brain regions in the current study indicates improved visual information integration in LPD patients and may further confirm its compensatory role in maintaining normal brain function, even normal motor function.

In our study, there were no evident differences in these regional metrics between RPD patients and HCs. Some researchers have proposed that RPD patients have better neural reserve and greater neural plasticity than LPD patients, and previous research has shown that patients with dominant-side onset exhibit a greater ability to cope with PD-related pathological changes than those with non-dominant-side onset (Ham et al., 2015; Chung et al., 2019). In the current study, all the subjects were right-handed. This may explain why patients with RPD did not markedly differ from HCs. The small sample size may be another potential factor underlying the lack of significant difference.

This study had some limitations. First, some patients with PD received dopamine treatment; although the treatment was terminated before MRI scanning, a possible effect of the drug cannot be completely ruled out. Second, the current analysis was not performed on different motor subtypes; it would have been more informative to do so. Finally, the current findings are based on a small sample; future studies should use larger sample sizes for in-depth exploration.

## Conclusion

In this study, we used rs-fMRI, DTI and FSH approaches to investigate the topological organization of the brain functional and structural connectome in PD with different sides of onset. The topological properties of the brain network differed between LPD and RPD. The nodal metrics may serve as potential imaging features to diagnose PD and monitor its progression. Collectively, these findings may provide neurobiological insights into the lateralization of the onset of PD.

## Data availability statement

The original contributions presented in this study are included in the article/Supplementary material, further inquiries can be directed to the corresponding authors.

## Ethics statement

The studies involving human participants were reviewed and approved by Xuzhou Affiliated Hospital. The patients/participants provided their written informed consent to participate in this study.

## Author contributions

XZ, CZ, and KX: research project conception. XZ, YX, and JX: research project organization and execution. HZ, RL, JS, QX, and CZ: statistical analysis design and execution. QX, RL, JX, and KX: statistical analysis review. XZ, YX, and LC: writing of the first draft. RL and KX: manuscript review. CZ and KX: take responsibility for the data. All authors contributed to the article and approved the submitted version.

## Footnotes

1
https://www.nitrc.org/projects/gretna/
