# Supplementary material for: Topological patterns of motor networks in Parkinson’s disease with different sides of onset: A resting-state-informed structural connectome study
Source: Front Aging Neurosci. 2022 Oct 26;14:1041744. doi: 10.3389/fnagi.2022.1041744 (PMC9643776; doi:10.3389/fnagi.2022.1041744)
Supplement: Supplementary file 1 [file Image_1.pdf]

# *Supplementary Material*

## Supplementary Figures

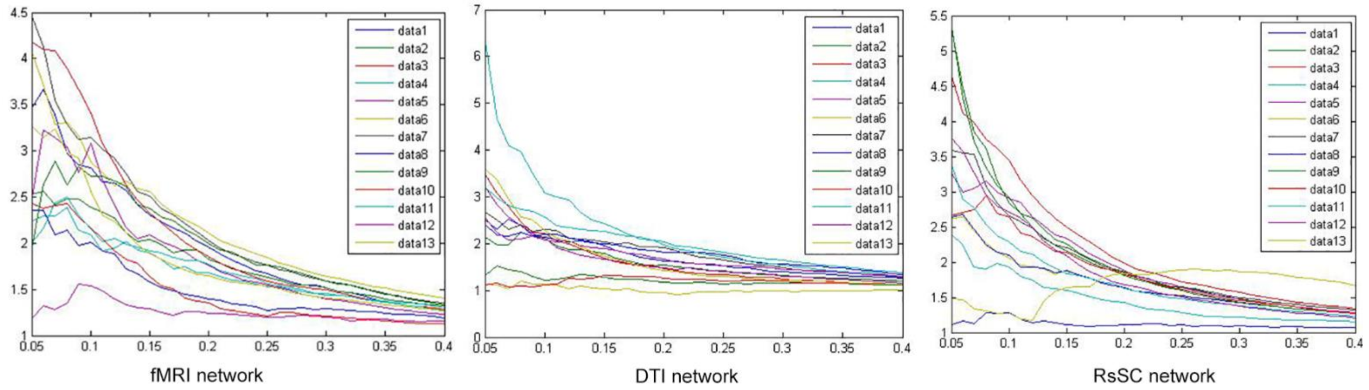


**Supplementary Figure 1.** All three groups of subjects (LPD patients, RPD patients, and HCs) showed small-world properties in fMRI network, DTI network and RsSC network.
